# Chromosomal instability triggers cell death via local signalling through the innate immune receptor Toll

**DOI:** 10.18632/oncotarget.6035

**Published:** 2015-10-10

**Authors:** Dawei Liu, Zeeshan Shaukat, Robert B. Saint, Stephen L. Gregory

**Affiliations:** ^1^ School of Biological Sciences, University of Adelaide, Adelaide, Australia

**Keywords:** chromosomal instability, innate immune response, Drosophila, TNFα, TLRs, Immunology Section, Immunity, Immune response

## Abstract

Chromosomal instability (CIN) is a hallmark of cancer and has been implicated in cancer initiation, progression and the development of resistance to traditional cancer therapy. Here we identify a new property of CIN cells, showing that inducing CIN in proliferating *Drosophila* larval tissue leads to the activation of innate immune signalling in CIN cells. Manipulation of this immune pathway strongly affects the survival of CIN cells, primarily via JNK, which responds to both Toll and TNFα/Eiger. This pathway also activates Mmp1, which recruits hemocytes to the CIN tissue to provide local amplification of the immune response that is needed for effective elimination of CIN cells.

## INTRODUCTION

Chromosomal Instability (CIN) refers to a state in which cells have an increased rate of gain or loss of whole chromosomes or chromosomal pieces [1]. Several causes of CIN have been identified, and include dysfunction of the spindle assembly checkpoint, centrosomes, DNA replication or cohesion, leading to defects in chromosome segregation during mitosis [1, 2]. CIN is a defining feature of most human solid cancers (e.g. 85% of sporadic colorectal cancers) and is positively correlated with both drug resistance and poor prognosis [3]. Somatic cells with CIN can initiate the process of tumorigenesis [4], and CIN is responsible for the generation of cells with varied genetic backgrounds, out of which drug resistance can develop. This is thought to contribute to relapses following traditional cancer therapies that appear to initially succeed: the therapy creates selection pressure on cancer cells which drives rapid genetic evolution as CIN generates diverse cells from which those with a selective advantage and drug resistance can arise [5].

In this context, the role of the immune system is a double-edged sword during tumorigenesis [6]. On one hand, tumor-related inflammation is thought to foster tumorigenesis by supplying molecules into the tumour micro-environment that promote angiogenesis, resistance to apoptosis, and metastasis of malignant cells [7]. On the other hand, the immune system can detect and eliminate incipient cancer cells: there is good evidence for cancer immune surveillance. For example, immunocompromised mice that lack mature lymphocytes show a higher frequency of spontaneous tumorigenesis by the age of 14-16 months [8]. The frequency of carcinogen-induced tumorigenesis is also much higher in immunocompromised mice than in immunocompetent controls [9]. Furthermore, clinical evidence shows that at least for some kinds of tumours, increased infiltration with activated T cells is correlated with a better prognosis [10, 11]. Overall, the capability of cancer cells to circumvent attack by the immune system has been recognized as a hallmark of cancer [12].

Chromosomal instability represents a striking difference between the tumour and stromal cells, which do not normally have CIN. Consequently, CIN represents an excellent immune target if it can be recognized. Although the immune system has been reported to be activated by DNA damage [13] and tissue dysplasia [14], little is known about *in vivo* responses to CIN. While screening for genes that are required for the death of CIN cells *in vivo*, we identified several immune signalling genes. We found that the induction of CIN not only activates a systemic response from immune tissues, but also triggers a local immune reaction in proliferating epithelial cells. Manipulation of immune signalling strongly affects the fate of these CIN cells. Altogether, our results showed that the immune system can detect and respond to CIN, and represents a critical feedback loop that is necessary to ensure the removal of defective cells that are a threat to the organism.

## RESULTS

### CIN leads to mitochondrial dysfunction, oxidative stress and cell death

**Figure 1:**
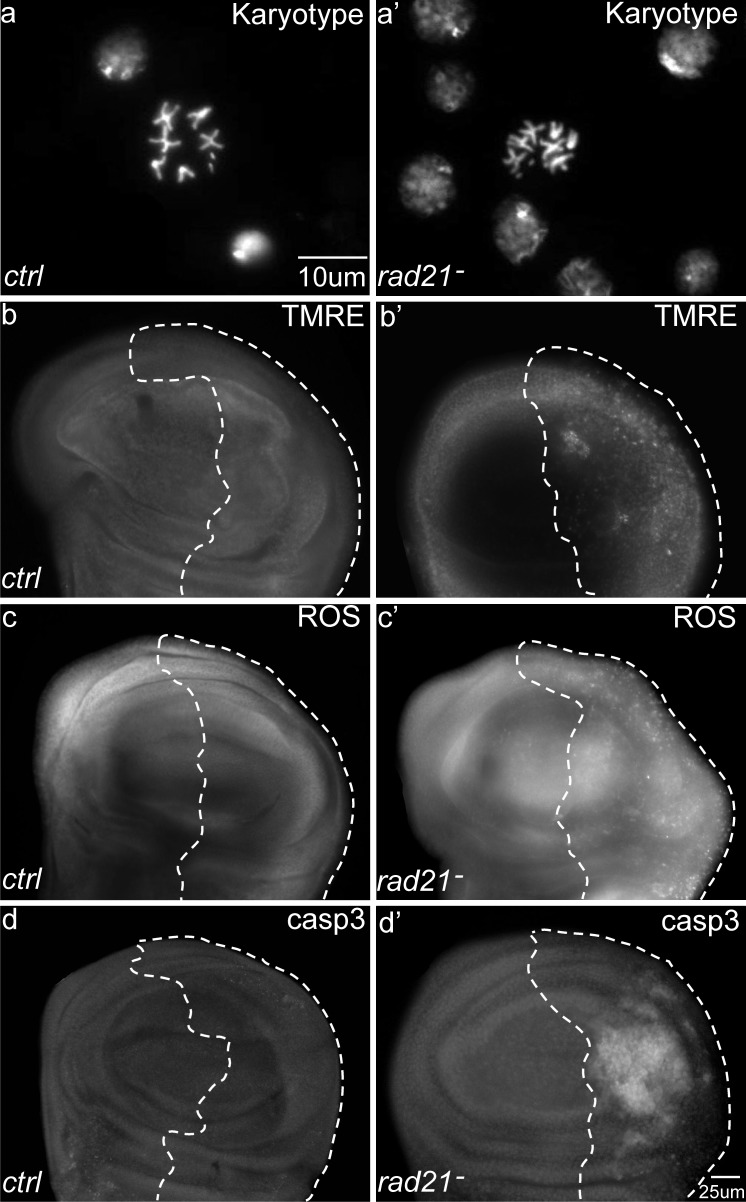
The effects of Chromosomal Instability (CIN) induced by Rad21 depletion on larval wing discs CIN was induced in the *engrailed* expressing posterior region of the wing discs as shown by the dotted line; the rest of each disc was wild type. (**a**, **a'**) DNA stains of metaphase cells to show the karyotype. (**a**) A normal karyotype. (**a'**) Karyotype from a wing disc cell with induced CIN (*engrailed*>*Gal4*, *UAS-rad21*^RNAi^ *UAS-Dicer2*) showing a chromosome gain. Aneuploidy rates were quantified in Fig S1. (**b**, **b'**) TMRE staining of third instar larval wing discs. Tissue with induced CIN (*engrailed*>*Gal4*, *UAS-rad21*^RNAi^ *UAS-Dicer2*) showed increased mitochondrial membrane potential (**b'**) compared to the negative control (**b**). (**c**, **c'**) CellRox staining of third instar larval wing discs. CIN cells showed increased oxidative stress (**c'**) compared to the negative control (**c**). (**d**, **d'**) Anti-cleaved caspase3 antibody staining of third instar larval wing discs. CIN tissue showed an increased level of apoptosis (**d'**) compared to the negative control (**d**).

We have previously reported that knockdown of the spindle assembly checkpoint gene *mad2* by RNA interference can be used to induce chromosomal instability (CIN) in *Drosophila* cells *in vivo*, which then show lagging chromosomes or chromosome bridges [15]. CIN caused by *mad2* knockdown leads to oxidative stress and a repair response from the JNK pathway [16, 17]. In order to generate higher levels of CIN and to confirm that these CIN phenotypes were not specific to *mad2* knockdown, we created another inducible-CIN model. We knocked down *rad21*, a cohesin that regulates sister chromatid separation during cell division [18, 19]. While Rad21 mutation is not common in advanced cancers, its depletion results in CIN in vertebrates [20]. Co-expressing Dicer2 to enhance the RNAi knockdown of *rad21* in proliferating wing imaginal disc cells resulted in aneuploidy in 46% of metaphase cells, indicating a relatively high rate of CIN (Figure 1a and Figure S1). To avoid missing cells that may have died from aneuploidy and been cleared, we blocked apoptosis by overexpression of p35 and in this case saw that around 70% of metaphase cells were aneuploid (Figure S1b). CIN induced by *rad21* depletion led to an increase in the level of TMRE staining, indicating elevated mitochondrial activity (Figure 1b). As expected, this was accompanied by an increased level of oxidative stress (Figure 1c) and widespread cell death (Figure 1d, Figure S1). These effects were consistent with, but stronger than the effects of *mad2* knockdown [16, 21]. We found that we could similarly increase the rate of aneuploidy and cell death in the *mad2* model by using temperature to increase the RNAi expression level or by blocking apoptosis (Figure S1b). These results indicated that chromosomal instability generated by disparate means resulted in mitochondrial dysfunction and oxidative stress. Using strong depletion of *rad21* or *mad2* we were able to generate high levels of instability making many cells inviable.

### Depletion of the toll pathway rescues lethality and apoptosis caused by chromosomal instability

**Figure 2:**
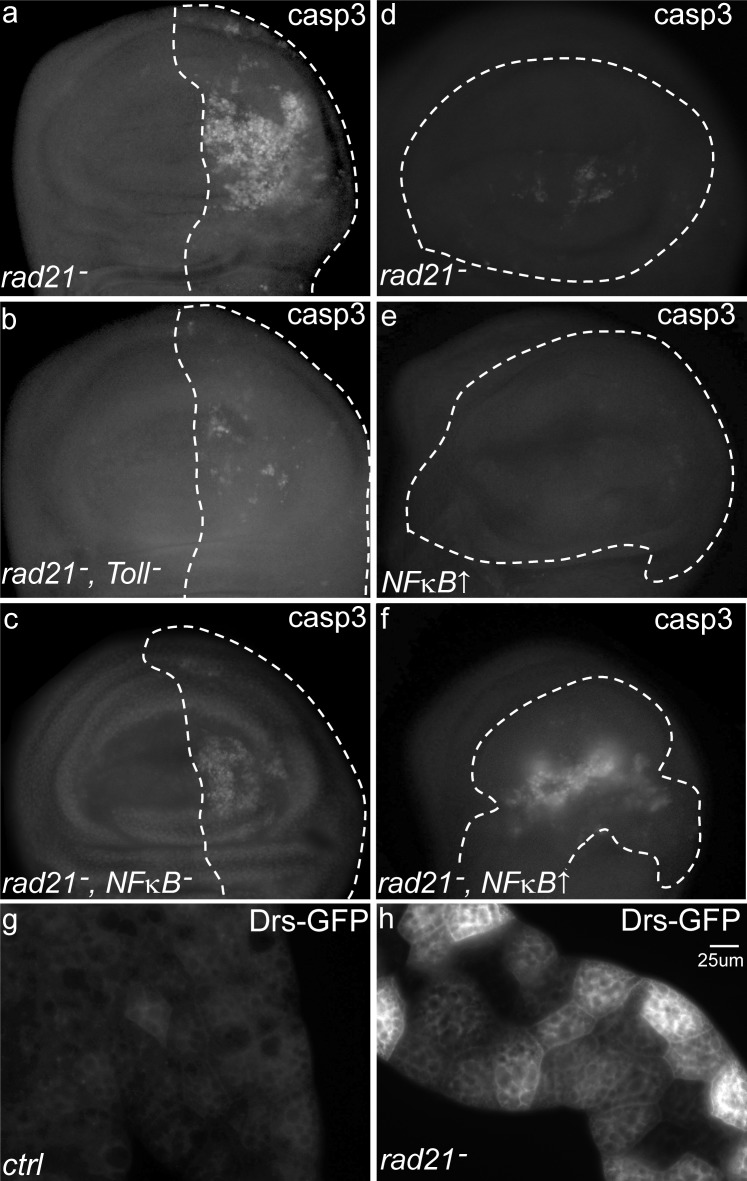
The effect of Toll pathway knock down or activation on CIN cell apoptosis Anti-cleaved caspase3 antibody staining shows apoptosis in third instar larval wing discs with CIN induced by depletion of Rad21 (*UAS-rad21*^RNAi^ *UAS-Dicer2*). (**a**, **b** and **c**) CIN and Toll pathway gene double knockdowns were induced in the *engrailed* region (driven by *engrailed*>*Gal4*) of the wing discs as shown by the dotted line; the remainder of each disc was wild type. Knocking down Toll (**b**) or NFκB (*dorsal*) (**c**) significantly reduced the level of apoptosis in CIN cells (**a**). (**d**, **e** and **f**) CIN and gene overexpression were induced in the dotted-lined region (driven by *MS1096*>*Gal4*) while the rest of each disc was wild type. Activation of the Toll pathway by NFκB (*dorsal*) over-expression caused little apoptosis in normal cells (**e**), but greatly increased the level of apoptosis in CIN cells (**f**). Note that the level of cell death induced by *Rad21*-RNAi was lower using the *MS1096* driver (**d**-**f**) than with the *engrailed* driver (**a**-**c**), allowing detection of CIN enhancement without killing the animal. Quantitation for these results is shown in Fig S2. The innate immune response from the primary immune tissue, the fat body, was detected by visualizing levels of a GFP-tagged antimicrobial peptide (Drosomycin-GFP) in the larval fat body (**g**, **h**). Wild type larvae expressed low levels of Drosomycin-GFP (**g**), but this level was increased in animals in which CIN had been induced in a range of tissues including the wing, epidermis, gut and fat body (h, *engrailed*>*Gal4*, *UAS-rad21*^RNAi^ *UAS-Dicer2*).

Having generated models in which high levels of CIN caused cell death, we were in a position to identify mechanisms that might be mutated in CIN cells (such as tumours) to improve their tolerance of this detrimental phenotype. Ubiquitous knockdown of *mad2* in *Drosophila* resulted in no adult survivors at 30°C, so we tested candidate gene knockdowns to identify any that could rescue this CIN lethality. While testing candidates involved in a variety of cellular processes, we found that knockdown of five *Drosophila* innate immune response genes from the Toll pathway could rescue the lethality in CIN flies (*Toll*, *dorsal*, *spatzle*, *cactus*, and *pelle*). These genes are part of a conserved signalling pathway that regulates patterning during early development and subsequently is used to mediate innate immune responses [22]. We next carried out cell death assays to examine whether the increased viability observed was due to a reduction in cell death when the Toll pathway was depleted in CIN cells. Knockdown of *Toll* or the NFκB homolog *dorsal* in CIN cells significantly reduced the rate of cell death as detected by Acridine Orange incorporation and anti-cleaved-caspase3 staining for apoptosis (Figure 2a-2c and Figure S2). We confirmed that the level of knockdown of *Mad2* was not decreased when we also knocked down *Toll* (Figure S1), excluding the possibility of Gal4 titration. Furthermore, we found that simulating Toll pathway activation by NFκB*/dorsal* overexpression greatly increased the level of apoptosis in CIN cells (Figure 2f) but had a limited effect on normal cells (Figure 2e). These results suggested that local activation of Toll pathway in CIN tissue is needed for the appropriate cell death response to high levels of CIN.

### CIN leads to an immune response

The Toll transmembrane receptor has been implicated in the induction of innate immune responses [23], so we hypothesized that the immune system could be activated in response to CIN. *Drosomycin* is an antimicrobial peptide gene that is a direct transcriptional target of Toll/NFκB signalling [24], so we used a Drosomycin-GFP reporter to detect its expression in the primary immune secretory tissue (fat body) of CIN larvae (Figure 2g, 2h). We observed a strong up-regulation of Drosomycin-GFP signal in 0 out of 12 control larvae and 11 out of 12 larvae with induced CIN. Together, these results suggest that induction of CIN leads to activation of the larval innate immune response. We also performed immunostaining against Dorsal and Relish, NFκB mediators of the *Drosophila* innate immune system downstream of Toll [25]. We found elevated levels of Dorsal in the cytoplasm of CIN wing disc cells (Figure S3). We observed a barely detectable increase in Relish (downstream of IMD) in CIN cells, even when using p35 to block apoptosis [26] and retain highly aneuploid cells (Figure S3).

### The immune system responds to reactive oxygen species

**Figure 3:**
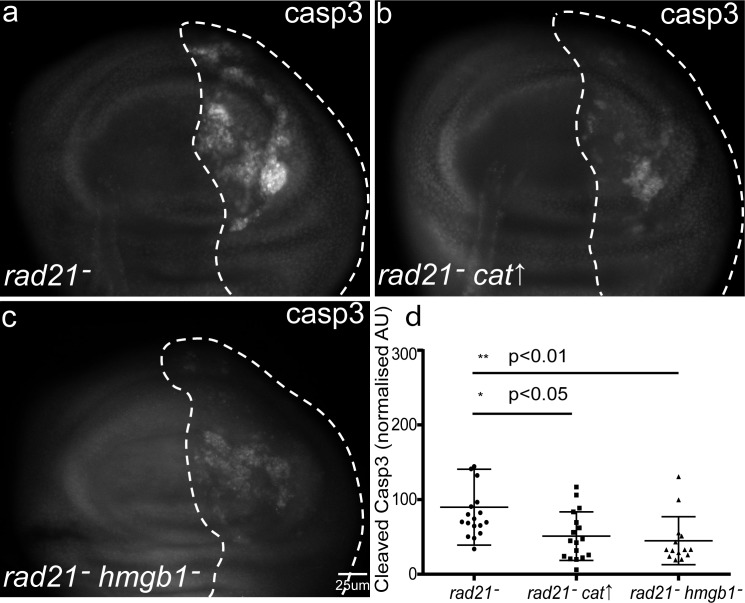
The role of reactive oxygen species (ROS) in determining the fate of CIN cells Anti-cleaved caspase3 antibody was used to detect apoptosis in third instar larval wing discs with CIN induced in the posterior (dotted) region (*engrailed*>*Gal4*, *UAS-rad21*^RNAi^ *UAS-Dicer2*) (**a**). Overexpression of Catalase to reduce oxidative stress (**b**) or knocking down the redox-sensitive damage marker HMGB1 (**c**) significantly reduced the level of apoptosis in CIN cells. (**d**) Quantification of the cleaved caspase3 staining. In all cases n≥10 and the error bars show 95% confidence intervals. The *p* values were calculated using two-tailed t-tests with Welch's correction.

Having found that the induction of CIN triggers an immune response we wished to understand what aspect of CIN cell biology is detected by the immune system. Reactive Oxygen Species (ROS) are known to activate both sterile and infectious inflammatory responses [27]. We have previously shown that CIN cells generate elevated levels of ROS [16], so we hypothesized that ROS might be a trigger. We found that over-expression of Catalase, which decreases ROS levels by converting H_2_O_2_ into H_2_O, significantly rescues the apoptosis observed in CIN cells (Figure 3b and Figure S4a-b). Knocking down the *Drosophila* ortholog of HMGB1 (Dsp1), a ROS-responsive effector of immune activation in vertebrates [27, 28] also rescued the apoptosis phenotype in CIN cells (Figure 3c). These results suggest a model in which the ROS generated by CIN cells is responsible for triggering an immune response. While there are likely to be many substrates affected by a ROS signal, the response may be mediated by the release of redox sensitive substrates like HMGB1 that are known ligands for the Toll pathway in vertebrates; the inflammatory response driven by Toll activation then significantly contributes to CIN cell death.

### Cell death caused by CIN is TNFα and JNK dependent

**Figure 4:**
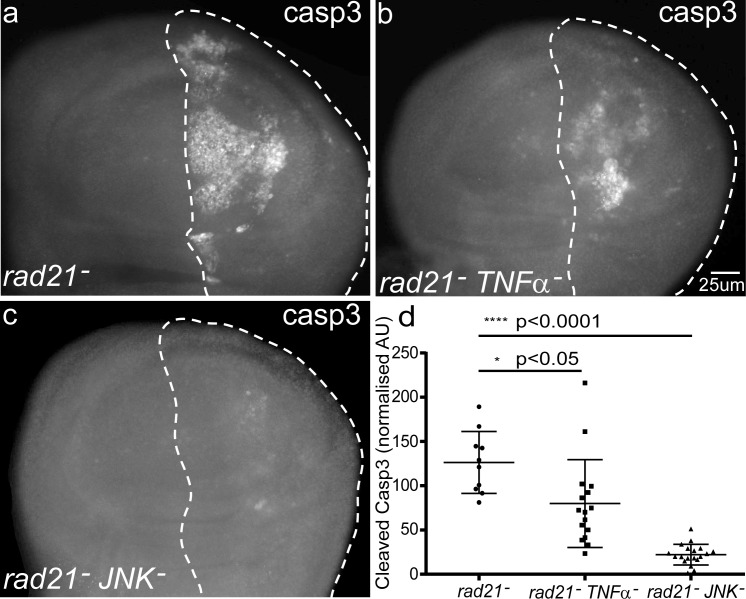
The effect of blocking TNFα signalling by depletion of Eiger or JNK, on the fate of CIN cells (**a-d**) Anti-cleaved caspase3 antibody was used to detect apoptosis in third instar larval wing discs with CIN induced in the posterior (dotted) region (*engrailed*>*Gal4*, *UAS-rad21*^RNAi^ *UAS-Dicer2*). Knocking down either TNFα (UAS-*eiger*^RNAi)^ (**b**) or JNK (UAS-*bsk*^RNAi^) (**c**) significantly reduced the rate of apoptosis in CIN cells. In these experiments TNFα production by immune cells such as circulating hemocytes was not altered; the knockdown was restricted to *engrailed*-expressing tissues such as the imaginal discs. Panel (**d**) shows quantification of the cleaved caspase3 staining. In all cases n≥10 and the error bars show 95% confidence intervals. The *p* values were calculated using two-tailed t-tests with Welch's correction.

**Figure 5:**
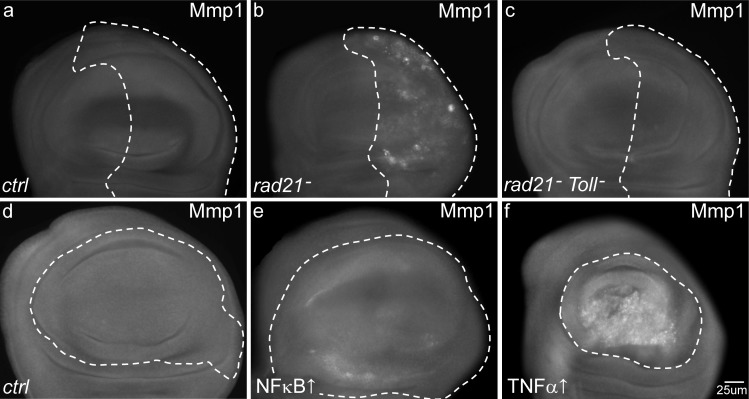
The effects of CIN and local immune signalling on the activation of matrix metalloprotease 1 Anti-Mmp1 antibodies were used to detect the levels of matrix metalloprotease 1 in third instar larval wing discs in which genes were knocked down (dotted regions) using *engrailed* (**a**-**c**) or *MS1096* (**d**-**f**) drivers. Normal wing discs (**a**, **d**) show low levels of Mmp1 staining. (**b**) When CIN was induced in the posterior region (dotted) of the disc (*engrailed*>*Gal4*, *UAS-rad21*^RNAi^ *UAS-Dicer2*), cells showed increased Mmp1 staining. Local signalling via Toll was needed for this effect, as Toll knockdown in these cells (**c**) reduced the level of Mmp1 staining in CIN cells. Overexpression of NFκB (*dorsal*) in the wing pouch (**e**, dotted region) led to a slightly increased level of Mmp1 staining. Overexpression of TNFα (*eiger*) in the same region (**f**) gave very high levels of Mmp1. *MS1096>Gal4* was used in these overexpression experiments to avoid lethality.

Toll signaling in *Drosophila* is known to generate a humoral response through antimicrobial peptides and to activate hemocytes that contribute to tumour clearance by TNF signalling [14, 29]. We tested whether Eiger, the *Drosophila* homolog of TNFα was also involved in mediating the apoptosis of CIN cells. Knockdown of TNFα/*eiger* by dsRNA in wing discs significantly reduced the apoptosis in CIN cells (Figure 4b). TNFα has been shown to cause cell death via the JNK pathway [30], so we tested the role of JNK in mediating the response to CIN. Knockdown of JNK strongly rescued the apoptosis of these CIN cells (Figure 4c). Looking downstream of JNK, we found that the JNK effector Mmp1 [31] was elevated in CIN cells (Figure 5b) but was lost if Toll signalling was reduced (Figure 5c). Overexpression of either TNFα or the Toll effector NFκB/Dorsal was sufficient to give elevated Mmp1 levels in normal wing discs (Figure 5e and Figure 5f), consistent with JNK and Mmp1 activation being downstream of Toll signaling. Our results show that Toll/NFκB signalling is needed in the CIN tissue itself for the TNFα-JNK mediated cell death usually seen when CIN is induced by *rad21* knockdown.

### A local immune response is critical for hemocyte recruitment

**Figure 6:**
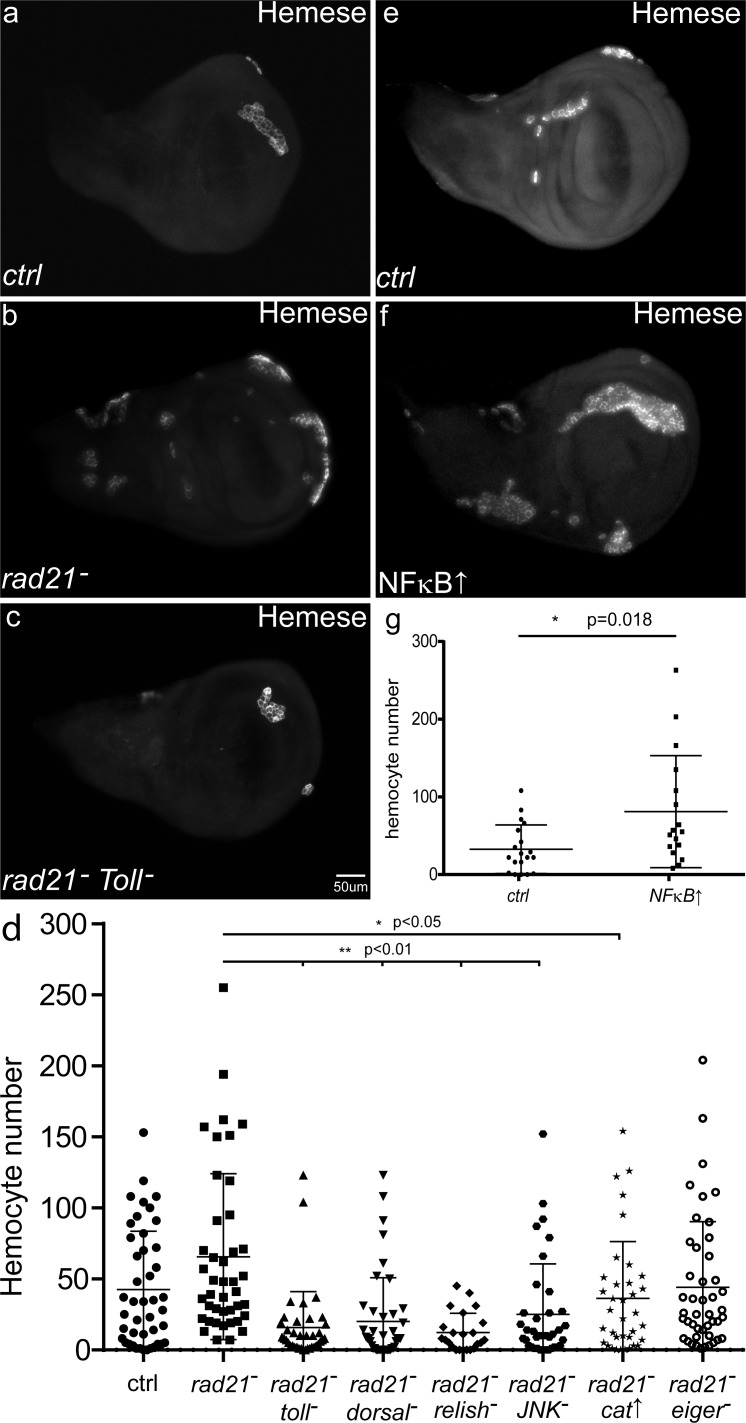
The effects of CIN and immune signalling on the recruitment of hemocytes Anti-Hemese staining was used to visualize the recruitment of macrophage-like hemocytes to the surface of third instar larval wing discs. When CIN was induced in the wing discs (*engrailed*>*Gal4*, *UAS-rad21*^RNAi^ *UAS-Dicer2*), significantly more hemocytes were recruited (**b**, **d**, *p* < 0.05) than to wild type wing discs (**a**, **d**). Blocking immune signalling in the CIN cells by Toll knockdown (**c**, **d**) greatly reduced the number of hemocytes recruited. (**d**, **g**) The quantifications show the number of hemocytes recruited to the wing discs, n≥20 in all cases, the error bars show 95% confidence intervals around the mean. (d) Knockdown of Toll, NFκB homologs (*dorsal* or *relish*), or JNK (*bsk*) strongly reduced the number of hemocytes recruited to CIN wing discs (*p* < 0.01for each). Overexpression of Catalase to reduce the level of oxidative stress generated by CIN cells also significantly reduced the number of hemocytes recruited to CIN wing discs (*p* < 0.05). Knockdown of *eiger* (TNFα) did not have a strong effect on the number of hemocytes recruited to CIN wing discs (*p* = 0.06). Simulation of local immune signalling by overexpression of NFκB (*MS1096*>*Gal4*, UAS-*dorsal*) in wing discs (**f**, **g**) was sufficient to significantly increase the number of hemocytes recruited compared to control discs (**e**, **g**). All p values were calculated by two-tailed t-tests with Welch's correction.

**Figure 7:**
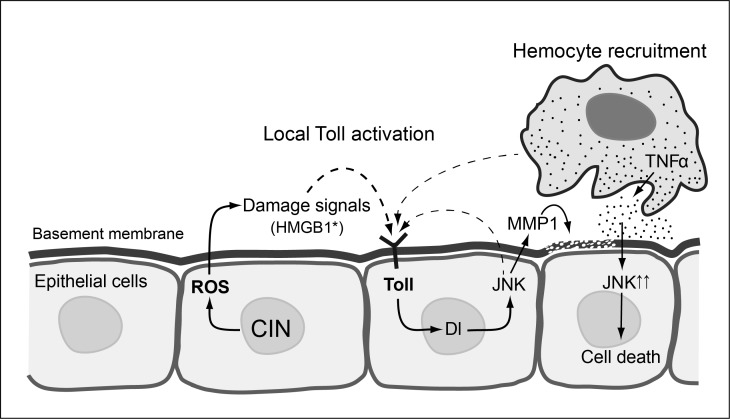
Proposed model for local Toll pathway activation giving apoptosis in response to CIN CIN cells generate ROS and DAMPs like oxidised HMGB1, which trigger the local immune response in nearby cells through the Toll/NFκB pathway which in turn activates JNK and Mmp1. The activation of Mmp1 leads to basement membrane digestion and hemocyte recruitment. The recruited hemocytes subsequently trigger the apoptosis of underlying cells through secretion of TNFα and further activation of JNK. Note that the signalling processes are drawn in adjacent cells for clarity, but may all occur in the same cell. Dashed lines indicate the production of activated Toll ligand by a process that is not well characterized. This model is informed by data from [34] and [14] on hemocyte responses and [65] on JNK activation of Toll.

One effect of activating the *Drosophila* innate immune response is the production and recruitment of hemocytes to sites of damage [32, 33]. Dysplastic or pre-tumorous tissue in flies can trigger this response, leading to increased numbers of hemocytes and recruitment of hemocytes to the surface of the abnormal tissue [14, 34]. We found that induction of CIN in otherwise normal, non-dysplastic tissue also increased the number of hemocytes recruited to the wing discs (Figure 6a-6b, 6d). Simulating local immune activation by NFκB overexpression was often sufficient to trigger the JNK-Mmp1 pathway and to recruit hemocytes (Figure 6e-6g). On the other hand, blocking local immune activation by Toll knockdown reduced the level of Mmp1 activation within CIN cells and lowered the number of hemocytes recruited to the wing discs (Figure 5c and 6c-6d). The same loss of hemocyte recruitment was produced by blocking JNK or either NFkB homolog, or by decreasing ROS levels (Figure 6d). These results suggest that a ROS-triggered local immune response in the wing disc is critical for hemocyte recruitment and the effective killing of CIN cells (Figure 7).

## DISCUSSION

As a feature of most human solid tumours, chromosomal instability (CIN) has been associated with the initiation of tumorigenesis [4], the development of drug resistance, and the poor prognosis of cancer patients after chemotherapy [35-38]. However, the induction of CIN in proliferating cells is usually detrimental or lethal, and the mechanisms by which cancer cells can tolerate CIN are poorly understood [39]. In order to investigate the signalling pathways that allow CIN tolerance, we carried out viability screening in *Drosophila* to select genes whose depletion could rescue lethality caused by CIN. Interestingly, five of the candidate genes obtained were from the *Drosophila* immune system. Further experiments showed that the depletion of several key genes in *Drosophila* immune pathways, such as *Toll*, *dorsal* and *relish*, could rescue the apoptosis phenotype caused by CIN in a proliferating tissue (Figure 2). These results strongly suggest that the innate immune system is normally induced to kill CIN cells.

Immune systems have long been thought to be involved in tumorigenesis [7, 40]. Chronic inflammation is thought to contribute to the initiation, promotion and progression of tumours [40]. However, the innate and adaptive immune systems may be able to eliminate transformed cells, so evasion of this immunosurveillance has been recognised as a hallmark of cancer [12, 41]. In this study, we showed that the induction of CIN in *Drosophila* larvae could trigger the production of antimicrobial peptides from the fat body, the main organ that drives humoral and cellular responses to damage and infection [24, 42]. This result is consistent with recent studies showing that induced tissue overgrowth in flies activates a systemic immune response [14, 42]. They also saw activation of the Toll pathway in the fat body, however they did not test the role of local immune signalling within the induced tumour.

In this study, we found that induction of CIN not only activated the immune organs, but also triggered an immune response within the proliferating CIN tissue (Figure S3). The Toll and Imd pathways are activated in CIN wing discs and depletion of either Toll or Dorsal just in the wing cells reduced the amount of apoptosis in response to CIN (Figure 2). Our data suggests a model in which the local immune response activates JNK and Mmp1 to recruit hemocytes which in turn trigger apoptosis in those cells (Figures 5 and 6). Consistent with this model, we have seen that enhancing the local immune response by *dorsal* overexpression greatly increased the level of apoptosis in CIN cells (Figure 2). On the other hand, blocking the local immune response and its downstream effectors by knocking down Toll, Dorsal, Relish or JNK could significantly reduce the number of hemocytes recruited (Figure 6d). Hemocytes have been shown to secrete TNFα onto the underlying tissue as well as being a source of the Toll ligand Spz [14], so hemocyte recruitment appears to be a positive feedback loop by which damaged cells attract a source of signals to ensure their own demise (Figure 7).

The activation of local immune signaling in proliferating tissue has been shown to remove slow growing cells [43]. In that case the trigger(s) and targeting are not known, and the Toll receptor itself and JNK had little effect. Thus, although cell competition presents an interesting parallel, it appears to induce a response different to that seen in CIN cells. Local as well as systemic immune responses are also seen in response to infection by bacterial pathogens [44], suggesting that the systemic response alone is either insufficient or requires targeting. For example, bacterial pathogen elimination in infection requires local IMD/Relish pathway activation [45-47]. At least in the gut, it appears that tissue damage and ROS production are used as an additional trigger to improve immune responses to pathogens [44]. In the case of CIN, we think that ROS are made in the mitochondria [16] rather than at the plasma membrane by Duox [48], but the immune effects may be similar.

Toll and Toll-like receptors (TLRs) are the critical mediators of innate immune responses in *Drosophila* and mammals [23, 49]. Apart from external pathogens, many endogenous molecules released from damaged cells, referred to as DAMPs (damage-associated molecular patterns), can also activate Toll-like receptors [6]. Toll has recently been implicated in the response to tissue dysplasia and damage [14, 50], suggesting a similarity to vertebrate DAMP receptor TLRs. However it should be noted that TLRs frequently detect the DAMP directly, while activation of the Toll ligand Spz is likely to require several intermediate steps [51]. Our results showed that CIN cells exhibit dysfunctional mitochondria and oxidative stress, both of which are known to activate innate immune responses through TLRs [27, 52]. Reducing oxidative stress by over-expressing the antioxidant Catalase significantly rescued the apoptosis phenotype of CIN cells. Consistent with this model in which ROS triggers an immune response, Catalase overexpression also significantly reduced the number of hemocytes recruited (Figure 6d). In addition, removing dysfunctional mitochondria by over-expressing the mitophagy gene *park1*, which mediates the clearance of abnormal mitochondria [53], also rescues the apoptosis phenotype of CIN cells (our unpublished data). It is not known how CIN generates stressed mitochondria, but current models for stable aneuploidy suggest that altered stoichiometry of proteins can lead to saturation of the protein folding and degradation machinery, leading to ER stress and subsequent mitochondrial stress [54]. We are confident that ROS are an upstream trigger rather than a downstream consequence of apoptosis, because decreasing ROS levels reduced apoptosis in CIN tissue (Figure 3b) and we were able to almost completely block apoptosis without reducing ROS levels (Figure S4).

In order to identify potential mediators of the ROS signal, we tested HMGB1 (high mobility group box 1), which is one of the most intensively studied DAMP molecules. HMGB1 is redox state sensitive and can be released from oxidatively damaged cells, triggering immune responses by binding to Toll-like receptors [27]. We found that HMGB1 depletion could rescue the apoptosis phenotype of CIN cells, suggesting a model in which ROS triggers a local immune response by releasing oxidised HMGB1, leading eventually to CIN cell apoptosis. Consistent with this model, research in mammals has shown that HMGB1 released from dying cells triggers a TLR4 dependent immune response that affects the outcome of traditional cancer therapy [55].

We expected that apoptosis in response to CIN would be mediated by the TNFα/Eiger-JNK pathway, which has been well documented to trigger apoptosis in flies in response to a number of stimuli [30, 56, 57]. Eiger is the Drosophila ortholog of Tumour Necrosis Factor alpha (TNFα) which acts as tumour suppressor and typically drives apoptosis by activation of the intrinsic death pathway though JNK [30, 56]. JNK signalling has been shown to be dysregulated in several fly “pre-tumour” models with varying effects including apoptosis, migration, proliferation and DNA repair. [14, 17, 31, 57, 58]. In CIN cells, we detected increased JNK signalling (Figure 5), and depletion of either *eiger* or *JNK* by RNAi could significantly rescue the apoptosis phenotype (Figure 4). These results are consistent with the role of the TNFα-JNK pathway as a tumour suppressor signal to eliminate CIN cells by triggering apoptosis. JNK activation would also be expected to promote hemocyte proliferation to increase the local TNFα signal, as observed in response to tissue overgrowth [14, 34]. JNK signalling through Mmp1 can also lead to invasive cell migration [31, 59] typically when apoptosis has been blocked by strong growth factor signalling. Despite the activation of Mmp1 in CIN cells, we have not observed any invasion or metastasis. We speculate that this could be due to apoptotic clearance and the local immune response restraining the invasiveness of CIN cells.

Based on our results, we have hypothesised that CIN cells produce dysfunctional mitochondria and oxidative stress; the generation of ROS and DAMPs such as HMGB1 then triggers a local immune response. This involves signalling through Toll to give JNK activation, which is known to generate signals that attract [34] and expand [14] the hemocyte population. The recruited hemocytes then promote the death of CIN cells through TNFα-JNK signalling as well as secreting Spz to increase Toll signaling (Figure 7). We also saw some reduction in cell death when TNFα was depleted just in the CIN cells, so we speculate that ROS can generate some local production of TNFα, as has been reported for eye discs [60]. However this more immediate route to cell death (ROS-TNFα-JNK) does not appear to be very effective in CIN cells, because without Toll and the involvement of an immune response to amplify the JNK signal, we saw very little CIN cell death (Figure 2).

Our results raise the question of whether such an anti-CIN immune response has clinical implications. Investigation of the innate immune system as a cancer treatment has been going on for more than a century. In the 1890s, Coley injected live bacterial cultures into cancer patients as a treatment to provoke the immune system with some success in treating certain cancer types such as soft tissue sarcoma and lymphoma [61]. Since the 1950s, bacteria-derived materials like polysaccharide have been investigated for cancer immunotherapy. Although the detailed mechanism of their anti-cancer effect is unclear, some of them have been approved for clinical use [6]. DNA damage or DNA repair mutations are known to provoke an innate immune response [41, 62], a response that is likely to also be seen in CIN cells, as they generate ongoing DNA stress [2, 63]. Our results have suggested that CIN cells are aberrant in a number of significant ways including glucose metabolism, mitochondrial output, ROS levels, JNK signalling, and DNA damage [15-17], and that some or all of these contribute to a signal that generates the local and systemic immune responses needed to eliminate the damaged cells. It remains to be seen to what extent this response can be exploited therapeutically.

## MATERIALS AND METHODS

### Drosophila stocks

The fly stocks used in this paper are as follows: *mad2*-RNAi (VDRC 47918), *Rad21*-RNAi (Bloomington #36786), *Eiger* (TNFα)-RNAi (VDRC108814), UAS-*catalase* (Bloomington #24621), *HMGB1*-RNAi (Bloomington #31960), *Drosomycin*-GFP [24], UAS-*p35* (Bloomington #5073).

Driver stocks: *daughterless* (*da*)-Gal4 for ubiquitous expression, *engrailed* (*en*)-Gal4 for gene expression in the posterior region of wing discs and *MS1096*-Gal4 for wing pouch expression, all from Bloomington *Drosophila* stock centre.

### Viability screening

Candidate genes were knocked down in the CIN background (*mad2* knockdown) to see their effect on the viability of CIN flies: UAS>*mad2* RNAi/CyO; *da*>Gal4/TM6 *tubulin*>Gal80ts × UAS>candidate-RNAi. The crosses were set at 30°C which was lethal for CIN flies crossed to negative controls.

### RNA purification and quantitative real-time PCR (qPCR) assays

Five third instar larvae from each genotype (in triplicate for each genotype) were chosen and washed in PBS and were quickly transferred and homogenised in cold Trizol reagent on ice and then stored at −80°C before processing as described [15]. Primers pairs used in this paper:

*mad2* F/R:GGCGACCAAAAACTGCATCA/GGTAAATTCCGCGTTGGAAGA
*rp49* F/R:ATCGATATGCTAAGCTGTCGCAC/TGTCGATACCCTTGGGCTTG

### Karyotype analysis

For measuring the level of aneuploidy, wing discs from third instar larvae were dissected out in PBS, and were incubated for 10 min in 0.5% sodium citrate solution. Then these discs were treated with 45% acetic acid for 2 min and 60% acetic acid for 1 min on a cover slip. Treated wing discs were squashed quickly between a coverslip and a slide and placed into liquid nitrogen. The cover slip was removed and the squashed discs were stained with Hoechst 33342 for 10 min and washed with PBST for 20 min before mounted in 80% glycerol. The karyotypes of different genotypes were compared using χ^2^ analysis to detect significant variation from the expected proportions of euploid and aneuploid cells.

### Cell death

Acridine Orange (AO) staining was used to measure the cell death in larval wing imaginal discs [15]. Third instar larvae were dissected in PBS for imaginal discs; the collected imaginal discs were incubated in 1mM AO for 2 mins then transferred to a slide after a brief wash. Then the treated imaginal discs were immediately mounted in PBS with a cover slip on before microscopy. The results of AO were normalized by subtracting the wild type region value from the test region value (eg. *engrailed*-driven region) as identified by UAS>*CD8-GFP* expression. The background noise of all images was subtracted in ImageJ using a rolling ball radius of 10 pixels.

### Oxidative stress assay

The level of reactive oxygen species (ROS) in CIN cells was measured by using the fluorogenic probe CellROX from Life Technologies. The third instar larvae were dissected in D22 media pH 6.8. Then the dissected imaginal wing discs were transferred into 5μM CellRox (in D22 media) for 15 mins; after this, the wing discs were quickly washed in PBS and fixed in 3.7% formaldehyde for 5 min then mounted in 80% glycerol for imaging.

### Mitochondrial stress

The level of mitochondrial stress in CIN cells was measured by using the fluorogenic probe TMRE from Life Technologies. Third instar larvae were dissected in PBS and transferred into 0.05 μM TMRE solution for 10 mins incubation and then washed in PBS for 10 mins. Then the treated imaginal discs were immediately mounted in PBS for imaging.

### Immunostaining

Immunostaining was used on dissected wing imaginal discs for different purposes. Third instar larvae were dissected in PBS for imaginal discs; the collected imaginal discs were fixed in 3.7% formaldehyde for 20 mins and then wash for 30 mins in 0.2% PBST (1×PBS+0.2% Tween). For anti-hemese staining, the fixation time was 4°C overnight, with no shaking through all the process. The fixed imaginal wing discs were then blocked in PBSTF (1×PBS+0.2% Tween+5% fetal calf serum) for 30 mins and stained with the primary antibody for 2.5 hrs (at room temperature) or overnight (at 4°C). After staining with the primary antibody, the wing discs were washed in PBSTF for 30 mins then transferred to a secondary antibody solution for 2.5 hrs at room temperature in the dark. After 30 mins washing in PBST, the wing discs were mounted in 80% glycerol-PBS.

The source and concentration of antibodies used in this paper are as follows: Rabbit anti-caspase3 (D175, 1: 100) from Cell Signalling; mouse anti-dorsal (7A4, 22 μg/ml) and mouse anti-MMP1 (14A3D2, 5.3μg/ml) from the Developmental Studies Hybridoma Bank; mouse anti-hemese (1.5μg/ml) [64].

The secondary antibodies used were CY3 anti-rabbit (1: 100), rhodamine anti-mouse (1: 200).

### Data analysis

All microscopy was done on a Zeiss Axioplan2 microscope. Axiovision software (Carl Zeiss), Adobe Photoshop, Adobe Illustrator and ImageJ were used for image processing and quantification. Statistical analysis was carried out in GraphPad Prism using either t-tests or χ^2^ tests as indicated.

## SUPPLEMENTARY MATERIAL FIGURES AND TABLE




