# Supplementary material for: Chromosomal instability triggers cell death via local signalling through the innate immune receptor Toll
Source: Oncotarget. 2015 Oct 10;6(36):38552–65. doi: 10.18632/oncotarget.6035 (PMC4770720; doi:10.18632/oncotarget.6035)
Supplement: Supplementary file 1 [file oncotarget-06-38552-s001.pdf]

Chromosomal instability triggers cell death via local signalling through the innate immune receptor Toll

Supplementary Material

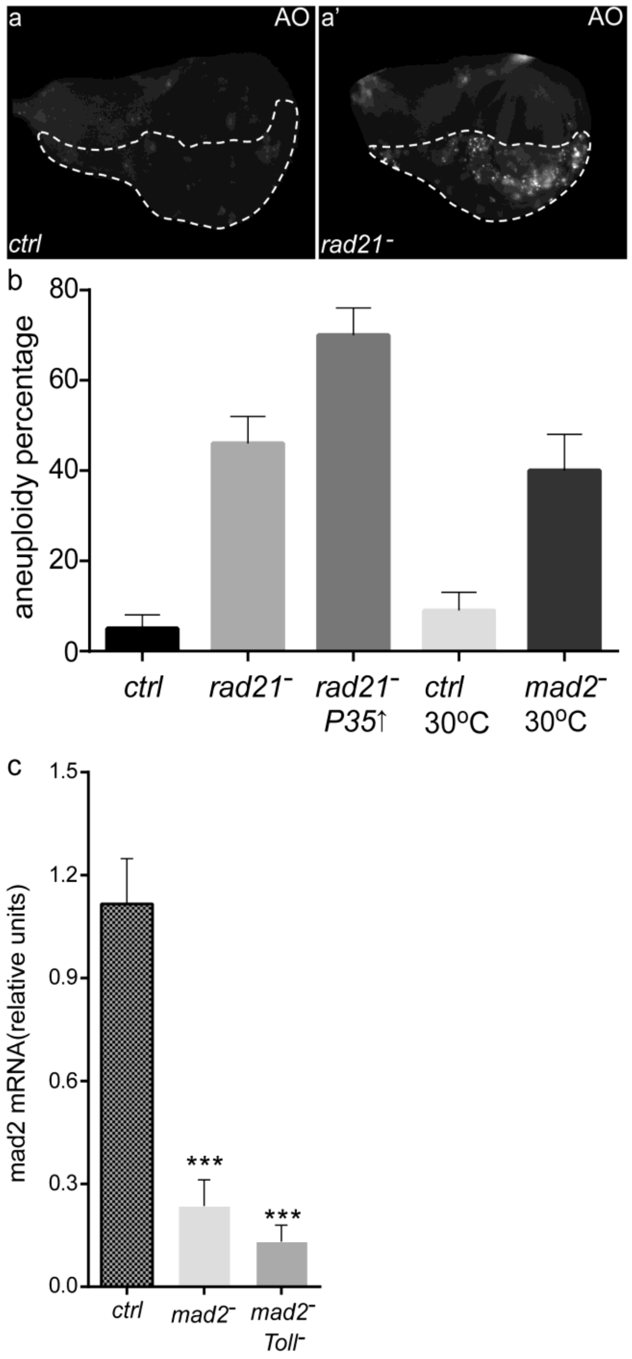

Fig S1 The effect of Chromosomal Instability (CIN) induced by Rad21 depletion on cell death in larval wing discs. (a, a’) Acridine Orange staining of third instar larval wing discs. CIN induced in the posterior (dotted) region of wing discs showed increased cell death (a’) compared to the negative control (a). (b) The level of aneuploidy in different CIN models. Metaphase karyotypes were used to evaluate aneuploidy as a proxy for the rate of CIN. *Rad21* knockdown in wing discs gave aneuploidy in 46% of metaphase cells while the level of aneuploidy increased to 70% if clearance of CIN cells by apoptosis was blocked by P35 over-expression. *Mad2* knockdown at 30℃ gave 40% metaphase aneuploidy. (c) The level of mad2 mRNA. qPCR shows that the ubiquitous expression of mad2 RNAi construct leads to mad2 mRNA reduction by 80%, and this knockdown effects is not affected by simultaneously toll knockdown indicating that more RNAi constructs do not affect RNAi machinery efficiency in UAS-Gal4 system.

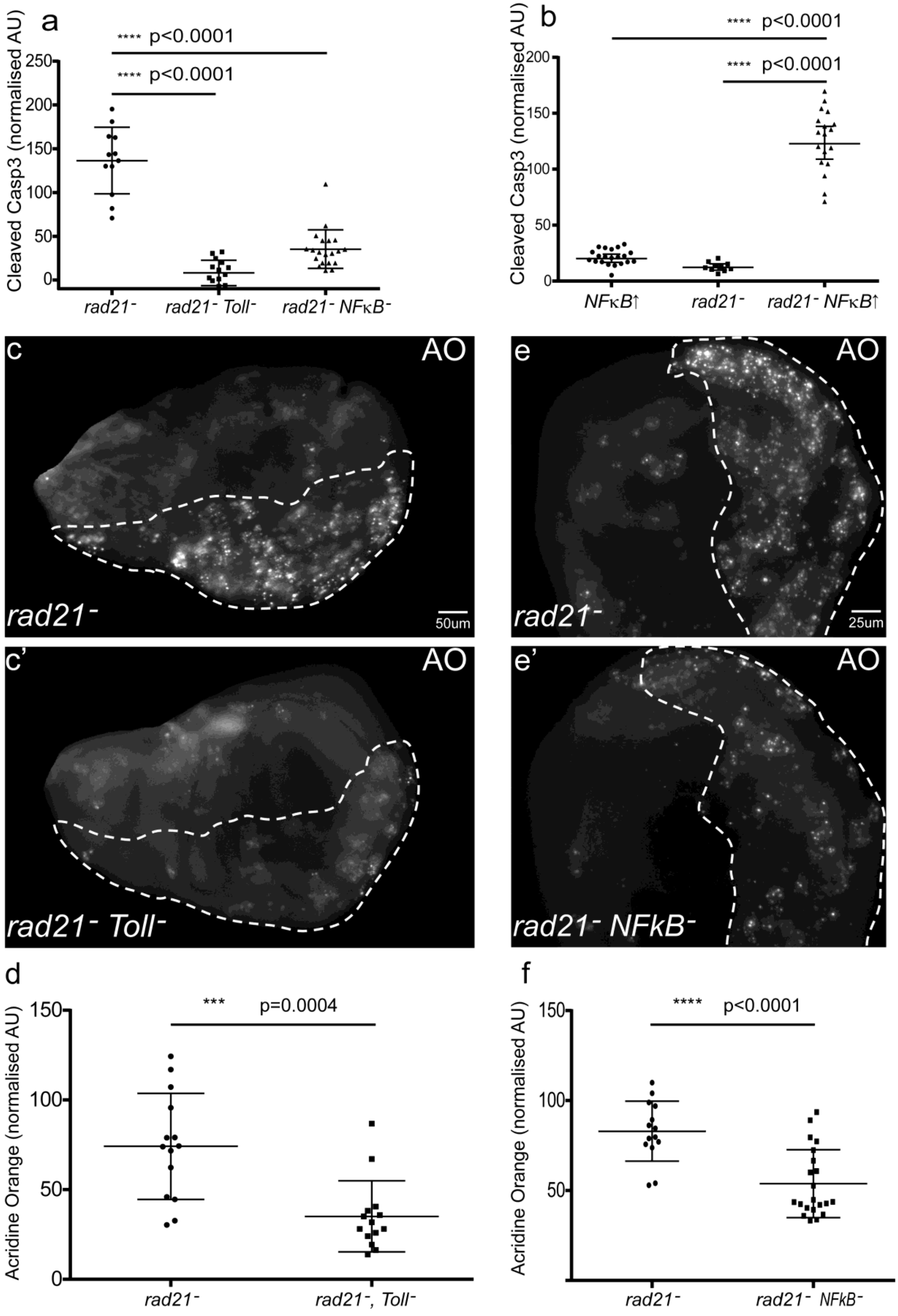

a
Cleaved Casp3 (normalised AU)
**** p<0.0001
**** p<0.0001
250
200
150
100
50
0
rad21⁻
rad21⁻ Toll⁻
rad21⁻ NFκB⁻
b
Cleaved Casp3 (normalised AU)
**** p<0.0001
**** p<0.0001
200
150
100
50
0
NFκB↑
rad21⁻
rad21⁻ NFκB↑
c
AO
rad21⁻
50um
c'
AO
rad21⁻ Toll⁻
e
AO
rad21⁻
25um
e'
AO
rad21⁻ NFkB⁻
d
Acridine Orange (normalised AU)
*** p=0.0004
150
100
50
0
rad21⁻
rad21⁻, Toll⁻
f
Acridine Orange (normalised AU)
**** p<0.0001
150
100
50
rad21⁻
rad21⁻ NFkB⁻

Fig S2 The effect of Toll pathway knockdown on CIN cell death.

(a and b) Quantification of the cleaved caspase3 staining in (Fig 2 a, b and c) and (d, e and f) respectively. The quantifications show the normalized grey value of staining that was obtained by subtracting the mean grey value of the wild type from that of the affected (dotted) region of each wing disc. n≥10 in all cases, the error bars show 95% confidence intervals. The p values were calculated using two-tailed t-tests with Welch's correction.

(c, c', e and e') Acridine Orange staining of third instar larval wing discs. Knocking down Toll (c') or NFκB (*dorsal*) (e') significantly reduced the level of cell death in CIN cells. (d, f) Quantification of Acridine Orange staining. The quantifications show the normalized grey value of staining obtained by subtracting the mean grey value of the wild type from that of the affected region of each wing disc. n≥10 in all cases, the error bars show 95% confidence intervals around the mean. The p values were calculated by two-tailed t-tests with Welch's correction.

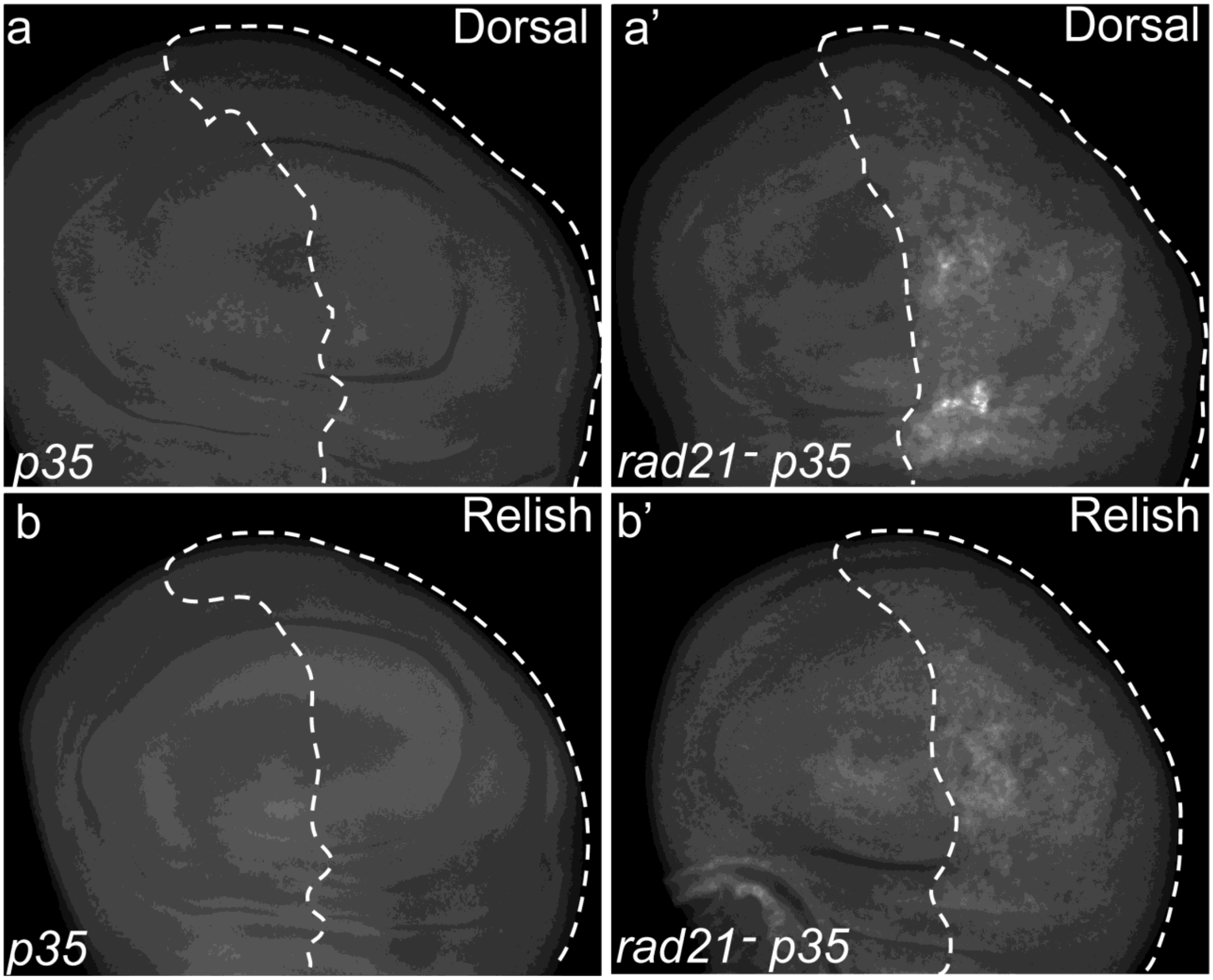

Fig S3 Visualizing activation of immune signaling by CIN.

(a, a', b, b') CIN was induced by depletion of Rad21 in the posterior half of third instar wing discs (*engrailed>Gal4*, *UAS-rad21*^{RNAi} *UAS-Dicer2 UAS-P35*), indicated by the dotted region. In this case, apoptosis was blocked by the expression of P35 to increase the retention of CIN cells that are otherwise cleared by apoptosis (see Fig 1). Activation of local immune signalling was detected by staining for the two NFkB homologs Dorsal (a, a') and Relish (b, b'). Levels of Dorsal were clearly elevated in CIN cells, with a barely detectable change in Relish. We did not observe any significant change in Dorsal subcellular localization; the Relish epitope detected would be expected to remain in the cytoplasm.

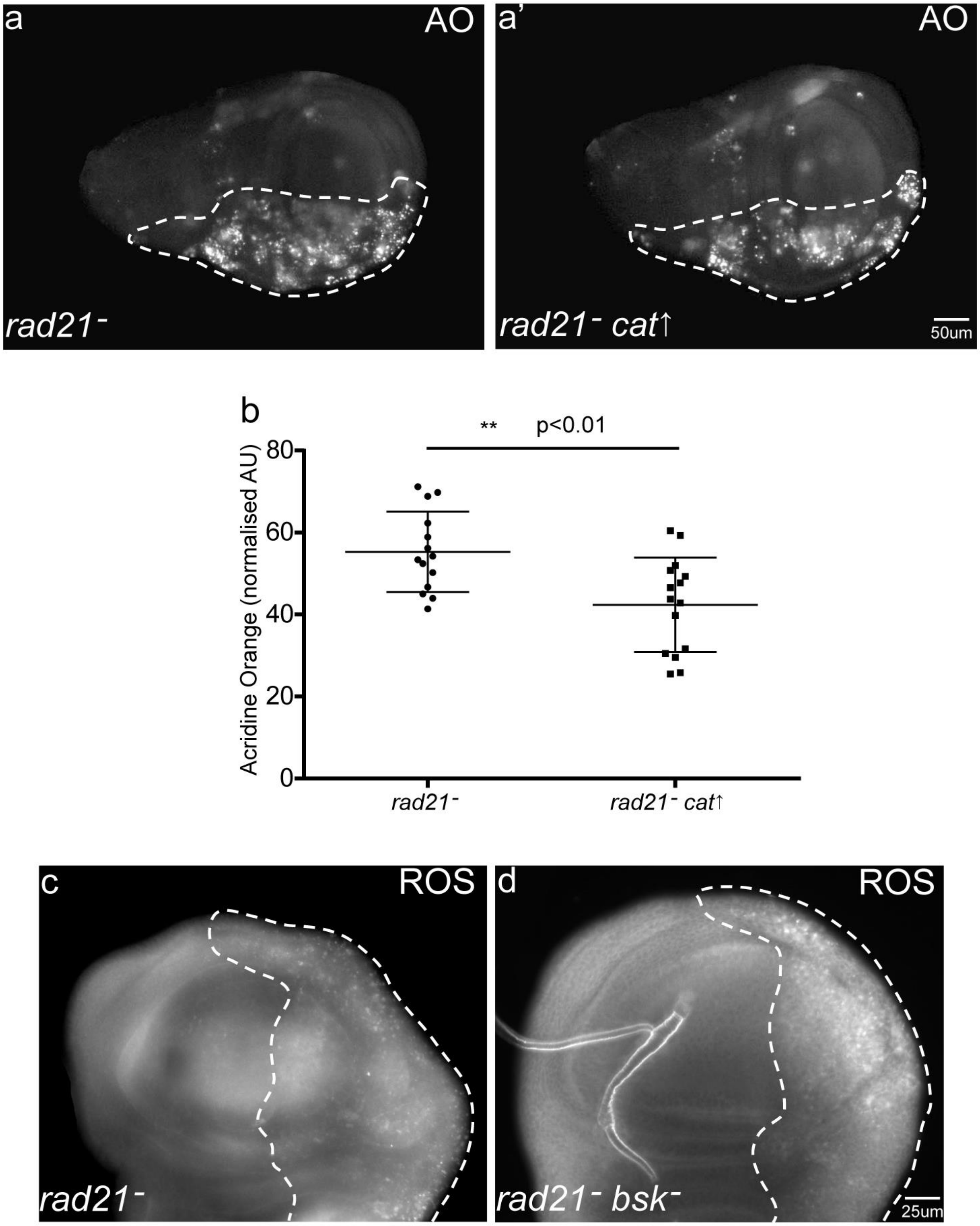

Fig S4 The role of reactive oxygen species (ROS) in determining the fate of CIN cells.

(a, a’) Acridine Orange staining of third instar larval wing discs. Overexpression of Catalase to deplete reactive oxygen species (a’) significantly reduced the level of cell death seen in CIN cells (a, b). (b) Quantification of the Acridine Orange staining with n>10 for each genotype, error bars showing 95% CIs and the p value calculated by a two-tailed t-test with Welch’s correction. (c) Reactive oxygen species, visualized here by CellRox staining, were elevated in CIN cells (dotted region) relative to the adjacent wild type tissue. (d) Blocking signalling by JNK (bsk^{RNAi}) did not reduce the level of reactive oxygen species in CIN cells, though it almost completely eliminated cell death (Fig 4). We concluded that ROS can cause cell death and is not being generated by the process of apoptosis.

Table S1

List of immune candidates showing rescue of *mad2* knockdown CIN induced lethality at 30°C. Columns show the ID of the RNAi construct used; its chromosomal location; the ID of the affected gene; the gene name; the number of surviving adults obtained when the candidate gene was knocked down in a wild type background and the number survivors when the candidate gene was knocked down in the CIN background (*da*>*mad2*). Obtaining surviving progeny indicated that the candidate gene knockdown rescued the *mad2* depletion lethality phenotype (marked in grey). Candidate lines that are unable to rescue the lethality are shown for comparison.
